# Editorial: Cyclic nucleotide phosphodiesterases (PDEs) signaling in the endocrine system

**DOI:** 10.3389/fendo.2025.1548972

**Published:** 2025-01-29

**Authors:** Federica Campolo, Arun Samidurai

**Affiliations:** ^1^ Department of Experimental Medicine, Sapienza University of Rome, Rome, Italy; ^2^ Departmental Faculty of Medicine, UniCamillus—Saint Camillus International University of Health Sciences, Rome, Italy; ^3^ Department of Internal Medicine, Division of Cardiology, Virginia Commonwealth University, Richmond, VA, United States

**Keywords:** cGMP, cAMP, PKA, PKG, endocrine system, endocrine diseases, phosphodiesterases (PDEs), PDEi

**Figure 1:**
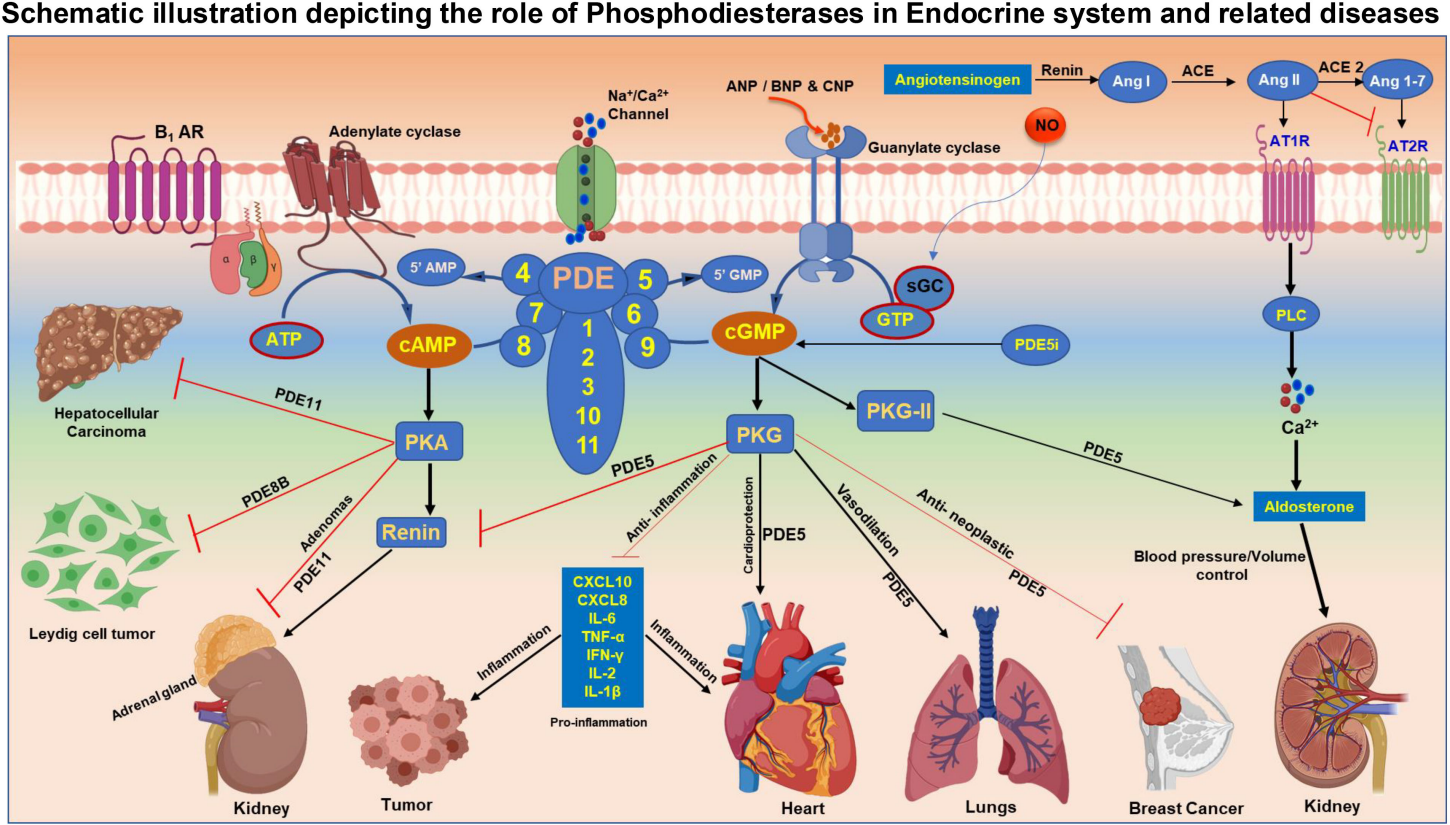
Figure-Schematic illustration depicting the role of Phosphodiesterases in Endocrine system and associated diseases. Adenylate cyclase (AC) activated by external stimuli such as hormones and insulin generate cAMP from ATP molecule. Activation of guanylate cyclase (GC) by ANP/BNP/CNP results in the synthesis of cGMP from GTP. The level of cAMP and cGMP second messengers in the cellular system is maintained by group of enzymes named phosphodiesterases (PDE). PDE enzymes are grouped based on their substrate specificity to cyclic nucleotides. PDE 4,7 and 8 exclusively hydrolyze cAMP, whereas PDE 5 ,6 and 9 are unique to cGMP degradation and PDE 1,2,3,10 and 11 are dual substrate specific that can catalyze the conversion of both cAMP and cGMP to AMP and GMP respectively. cAMP acts through protein kinase A (PKA), while cGMP executes the function predominantly via downstream target protein kinase G (PKG). Inhibition of PDE5 enhances the level of cGMP and offers cardioprotection and improves vasodilation in pulmonary arterial hypertension. PDE5 maintains blood pressure and fluid balance in the body through regulation of renin angiotensin-aldosterone system (RAAS). Induction of PDE5 results in tumor progression and PDE5i promotes apoptosis and impedes cell proliferation and enhances anti-inflammation. Abundant expression of PDE8 was noted in Leydig cell tumors (LCTs) and was involved in spermiogenesis and Leydig cell transformation. Germ line mutations in PDE 8 and PDE11 causes disruption in cAMP-PKA signaling pathway causing hepatocellular carcinoma (HCC) and adrenal gland adenomas.

Cyclic adenosine monophosphate (cAMP) and cyclic guanosine monophosphate (cGMP) are critical second messengers involved in numerous cellular functions, including metabolism, the endocrine system, and cancer (1). The level, compartmentalization, and amplitude of cAMP/cGMP responses are finely regulated by phosphodiesterases (PDEs) that are responsible for the hydrolysis of cyclic nucleotides in a spatio-temporal manner (2). PDEs are classified into 11 families (PDE1– PDE11) based on their specificity for cyclic nucleotides, structural homology, and mechanism of regulation (1). The PDE family of enzymes are encoded by more than 20 genes and undergo extensive splicing processes, giving rise to more than 100 different isoforms that are widely expressed in mammalian tissues (2, 3). PDEs govern the cellular levels of cAMP and cGMP and execute their function through their downstream protein kinase A (PKA) and protein kinase G (PKG) respectively. While PDE inhibitors (PDEi) have been successfully used for the treatment of many disorders, including erectile dysfunction (ED), pulmonary arterial hypertension (PAH), and lower urinary tract disease (LUTD), new evidence suggests a possible use of PDEi for the treatment of cardiovascular diseases (CVD), cancer, and metabolic disorders (4–8). Given the pivotal role of cyclic nucleotides in regulating many aspects of endocrine system physiology, pharmacological enhancement of cAMP/cGMP through PDEi has also been considered a valuable strategy for the treatment of endocrine disorders. Both cAMP and cGMP actively participate in the regulation of the endocrine system modulating hormone release in several endocrine tissues. The first evidence of the involvement of cGMP in the regulation of hormone release comes from the pioneering research of McKee et al., who reconstructed the intricate signaling governing hormonal release at the renal level, which involves nitric oxide and cGMP (9). In the hypothalamus, cGMP can stimulate the release of gonadotropin-releasing hormone, which is essential for reproductive function (10). cGMP signaling interacts with other signaling cascades, including cAMP and calcium signaling, to fine-tune endocrine responses. This cross-talk allows for complex integration of various signals and contributes to the precise control of hormone action (11). Like and more than its cognate, cAMP regulates the production and release of various hormones. In the adrenal cortex, cAMP stimulates the synthesis and secretion of cortisol in response to ACTH (12). Similarly, in the thyroid gland, cAMP mediates the effects of TSH on thyroid hormone production (13). For instance, in the pituitary gland, cAMP can stimulate cell proliferation and hormone production (14). These observations suggest that different cellular and molecular alterations of the cAMP-signaling pathway have been identified in endocrine-related diseases. Changes in cAMP signaling pathways have also been linked to tumorigenesis at different levels. The contribution of Bolger et al. deeply analyzes the role of cAMP signaling in cancers, highlighting its dual role as both a tumor promoter and suppressor depending on the cellular context. This duality is crucial for understanding the complexity of cancer biology and the potential for therapeutic interventions targeting the cAMP pathway. cAMP signaling is known to influence different cellular processes, including apoptosis, migration, and DNA repair mechanisms, which are critical in cancer progression and treatment responses. The complexity of cyclic nucleotide signaling in tumorigenesis stems from the wide variety of signaling pathways involved, the temporal and spatial specificity of their actions, tumor heterogeneity, the redundancy of many pathways, and the interaction with other cellular alterations. This complexity, combined with the difficulty in selectively manipulating cyclic nucleotide pathways *in vivo*, makes it challenging to fully dissect the molecular mechanisms by which cyclic nucleotides regulate tumorigenesis. A valuable contribution to understanding the functional role of cyclic nucleotide in regulating cancer endocrine-related disorders comes from the observations of Campolo et al., who have investigated the expression patterns and functional roles of PDE8 isoforms in human testicular tissues and Leydig cell tumors. The findings of their research highlight the significance of these isoforms in regulating cAMP levels, which are crucial for steroidogenesis in Leydig cells. Using an integrated translational approach, they emphasized the distinct roles of PDE8A and PDE8B in human testis and Leydig cell tumors, highlighting their potential as biomarkers and therapeutic targets in reproductive health and oncology. While PDE8 exclusively degrades cAMP, PDE5 is one of the major isoforms that selectively break down cGMP. Pharmacological inhibition of PDE5 results in potent vasodilation and hence PDE5i are successfully used in the treatment of ED (15), sickle cell disease (16), and PAH (17). Emerging studies also suggest that PDE5 plays an important role in inflammation and cancer. A review by Paronetto et al. elaborates on the role of PDE5 in inflammation associated with CVD and various cancers. Elevated levels of circulating chemokines such as CXCL10 and CXCL8 manifest in diseases like diabetes, metabolic syndrome, and cardiomyopathies that can contribute to vascular remodeling and heart failure. Results indicate that inhibition of PDE5 with sildenafil attenuates inflammation through stabilization of NO-cGMP signaling and reducing pro- inflammation cytokines such as IL-6, TNF-α, IFN-γ, IL-2, and IL-1β (18). PDE5 also enhances stromal fibroblast differentiation and secretion of chemokines CXCL16, which encourages the progression of cancer. Recent findings suggest that PDE5 plays an increasingly important role in prostate cancer (19, 20), breast cancer (21), colorectal cancer (22), brain cancer (23), and lung cancer (24, 25). The expression of PDE5 is induced in several cancer types, leading to inactivation of the cGMP-PKG signaling cascade. Therefore, blunting PDE5 induction in these scenarios is shown to promote apoptosis and suppress tumor growth by regulating cell proliferation. PDEs also play an important role in the regulation of the renin-angiotensin-aldosterone system (RAAS), a key endocrine mechanism that controls blood pressure and fluid balance in the body (26, 27). In this Research Topic, a review article by Gambaryan et al. summarizes the role of PDE in the RAAS. Renin secreted by Juxtaglomerular (JG) cells and aldosterone produced by Zona glomerulosa (ZG) cells are influenced by the cellular levels of cAMP and cGMP and hence PDEs (29). Regulation of RAAS by PDEs is highly complex and involves intriguing cross talk between cAMP and cGMP signaling orchestrated by PDEs. The secretion of renin is predominantly mediated by the cAMP/PKA pathway involving exchange proteins activated by cAMP (EPAC) (28). However, the role of cGMP in the activation of renin remains unclear and studies suggest that cGMP could both activate as well as inhibit renin. Several members of the PDE family are involved in RAAS signaling, including PDE1, PDE2, PDE3, PDE4 and PDE9, which are expressed in JG cells. Similarly, PDE2, PDE3, PDE8, and PDE11 are present in ZG cells, and their inhibition was shown to influence the level of aldosterone (29). PDEi, especially cilostazol (PDE3i), apremilastis (PDE4i), and sildenafil (PDE5i), are proven to be safe with minimal side effects. Therefore, repurposing PDE inhibitors for other diseases such as cancer, Leydig cell tumors, and hypertension may be a smart strategy. This Research Topic highlights the diverse role of PDEs in various diseases pertaining to the endocrine system associated with cancer and CVDs (**Figure 1**). Future directions using cutting-edge technologies like single-cell spatial transcriptomics may shed new light on the role of PDEs in the regulation of compartmentalized cAMP/cGMP signaling.
